# Eye-Movement Suppression in the Visual World Paradigm

**DOI:** 10.1162/opmi_a_00157

**Published:** 2024-08-15

**Authors:** Anna Laurinavichyute, Anastasia Ziubanova, Anastasiya Lopukhina

**Affiliations:** Department of Linguistics, University of Potsdam, Potsdam, Germany; Department of Psychology, Royal Holloway, University of London, UK

**Keywords:** eye-movement suppression, visual world paradigm, language-mediated eye movements, task demands

## Abstract

Eye movements in the visual world paradigm are known to depend not only on linguistic input but on such factors as task, pragmatic context, affordances, etc. However, the degree to which eye movements may depend on task rather than on linguistic input is unclear. The present study for the first time tests how task constraints modulate eye movement behavior in the visual world paradigm by probing whether participants could refrain from looking at the referred image. Across two experiments with and without comprehension questions (total *N* = 159), we found that when participants were instructed to avoid looking at the referred images, the probability of fixating these reduced from 58% to 18% while comprehension scores remained high. Although language-mediated eye movements could not be suppressed fully, the degree of possible decoupling of eye movements from language processing suggests that participants can withdraw at least some looks from the referred images when needed. If they do so to different degrees in different experimental conditions, comparisons between conditions might be compromised. We discuss some cases where participants could adopt different viewing behaviors depending on the experimental condition, and provide some tentative ways to test for such differences.

## INTRODUCTION

Introducing the visual world paradigm, Cooper (1974) demonstrated that while listening to stories, “… [participants] tend to spontaneously direct their line of sight to those elements which are most closely related to the meaning of the language currently heard” (Cooper, 1974, p. 84). The tight temporal association between the eye movements and the linguistic input that Cooper observed in the data suggested that eye movements could be used to tap into ongoing language processing.

At the same time, language comprehension is just one of the processes reflected in the eye movements along with visual information uptake, response planning and execution, etc., and does not necessarily have any preferred status (Degen et al., 2021; Falandays et al., 2020; Hayhoe & Ballard, 2005; McMurray, 2023; Yoon & Brown-Schmidt, 2018). The key role of individual’s goal in guiding eye movements has been known since the beginning of oculography, when Yarbus demonstrated that viewing the same image with different tasks produced entirely different viewing patterns (DeAngelus & Pelz, 2009; Yarbus, 1967). With regard to language processing, according to Salverda et al. (2011), the listener’s goal also takes precedence over language-mediated eye movements reflecting moment-to-moment processing. The demands of the task at hand, such as selecting or moving an object on the screen, can override the tendency to fixate the referred objects. Consequently, when a referent is irrelevant to the listener’s current goal, fixations on it will be greatly reduced, if at all present, and this should speak neither for nor against successful language processing.

For the visual world paradigm, the referential priority account (Knoeferle & Crocker, 2006; Knoeferle & Guerra, 2016) postulates that fixations on the object increase the most when a word refers to it directly. However, in case of a conflict between lexical and discourse pressures, eye movements tend to be more discourse-driven (i.e., goal-driven). Contrary to the referential priority account, participants do not look at the currently referred object if the broader discourse refers to another object (even without explicitly naming it). For example, in a study by Eberhard et al. (1995), participants heard “Put the saltshaker on the envelope in the bowl” while seeing a display with either one or two saltshakers (one was always on the envelope), an empty envelope, and an unrelated distractor. When hearing “on the envelope” and seeing one saltshaker, participants looked at the empty envelope in 55% of cases but only in 17% of cases if there were two saltshakers. Just hearing “the envelope” did not guide fixations to the respective image equally in both conditions; fixations increased when the envelope was perceived to be the goal of the planned action. In a similar vein, Sekerina et al. (2019) demonstrated that participants who heard stories about a boy pushing a girl at school, when asked “Who pushed the girl at school?” looked at the image of a boy (the correct response) more than at the explicitly mentioned girl or school while hearing the question.

The goal of the present study is to directly test to what degree task demands influence the viewing pattern in the visual world paradigm. To do so, we test an extreme case of inhibitory control task, i.e., whether eye movements in the visual world paradigm can be suppressed. We argue that the answer to the question might be important not only for theory but also for certain cases of practical use of the visual world paradigm. If participants can suppress the looks at the referred images to a great degree, at least some types of conclusions about language processing should be drawn with care.

This paper will focus on a certain type of visual world experiments comparing eye movements across different groups, such as native speakers and second language learners, or across conditions of varying difficulty. Consider the following hypothetical example: A study tests whether two groups of speakers can process a certain linguistic marker, such as accusative case in a language with obligatory case marking. Participants see two images: Either a bear is watching a rabbit, or the rabbit is watching the bear. The images are accompanied by sentences in one of two conditions: “The bear_NOM_ is watching the rabbit_ACC_” or “The rabbit_ACC_ is watching the bear_NOM_”. The task is to identify which image corresponds to the description that participants hear. Suppose that in one group, there are fewer fixations on the target image and/or they occur later than in the other group. In such a case, researchers may conclude that processing in this group is delayed and/or less accurate. In the extreme case, if the participants look at both images equally often, researchers may even conclude that these participants cannot process the case marking and therefore do not understand the sentence. Importantly, such conclusions are only warranted if eye movements directly mirror participants’ interpretation of the sentence. If they do not, then delayed and/or decreased fixations on the correct image do not necessarily reflect delayed and/or deficient morphosyntactic processing. In the Discussion section, we will address the question what delayed and/or decreased fixations could alternatively reflect.

The scenario outlined above is not merely hypothetical: Behavioral measures and neurophysiological markers that supposedly tap into the same processes can disagree. In beginner second language learners, a robust P600 response to ungrammatical sentences goes hand-in-hand with at-chance performance in the explicit grammaticality judgment task (Tokowicz & MacWhinney, 2005). Similarly, second language learners show sensitivity to non-words in their ERP response but perform at chance in the overt judgment task (McLaughlin et al., 2004). The dichotomy is not restricted to second language learners: When confronted with word category violations, native speakers exhibit an early negative ERP response even when violations are not consciously detected (Batterink & Neville, 2013; Rohaut & Naccache, 2017; van Gaal et al., 2014). Similar dichotomies also characterize visual processing: The activity recorded in the visual cortex when viewing masked images allows a deep neural network to classify the images as living/nonliving even when participants themselves perform at chance in the classification task (Mei et al., 2022). Taken together, these results show that behavioral responses may not faithfully reflect underlying processing, and, in particular, make it appear less effective. Could eye movements be closer to a mediated behavioral response, such as grammaticality judgment, than to a more unconscious index of processing, such as the ERP or fMRI signals?

To answer this question, we need to consider what processing mechanisms link language comprehension and eye movements in the visual world paradigm. The core assumption is that language processing influences visual attention allocation, which, in turn, increases the likelihood of fixating the attended object. However, visual attention allocation does not necessarily need to drive fixations. In fact, covert attention, i.e., deliberately shifting attention without performing a saccade to the attended area, has been widely studied in psychology (Carrasco, 2011; Zhao et al., 2012) and reading research (Engbert et al., 2002, 2005; Reichle et al., 2009; Reilly & Radach, 2003; Snell et al., 2018), but largely left out of the scope of visual world studies.

To the best of our knowledge, only two studies demonstrated covert attention in viewing tasks related to language processing. Salverda and Altmann (2011) (see also Soto & Humphreys, 2007) instructed participants to maintain their gaze on the fixation cross and to detect a slight change in the position of one of the two objects located to the left and to the right of the cross. While performing the detection task, participants also heard words that they were instructed to ignore. Participants responded fastest when they heard the name of the object that shifted, with intermediate speed when they heard the name of the object that was not on the screen, and slowest when they heard the name of the object that did not shift. These results demonstrate that even linguistic input that is known to be irrelevant to the task affects the allocation of visual attention. Crucially, the shift of visual attention did not initiate a saccade to the attended area: Participants maintained their gaze on the cross while attending to the object in 90% of all trials—that is, visual attention and eye movements could be decoupled to a high degree.

The outcomes of the study by Salverda and Altmann (2011) suggest that the typical reasoning used in visual world experiments may be problematic. Traditionally, researchers reason about attention allocation based on eye movements: If the image is fixated, its referent should be in the focus of attention. But if the overt eye movements can be successfully decoupled from covert visual attention, this reasoning holds only if researchers can prove that the degree of coupling between covert and overt attention was the same across compared groups and conditions. At the same time, Salverda and Altmann (2011) tested isolated out-of-context word processing, which reflects phonological processing and lexical access, but the effects may not necessarily extrapolate to the higher levels of language organization, such as sentence and discourse processing. The key question is, therefore, in the visual world setting, to what degree can eye movements in the visual world paradigm be suppressed?

To answer the question, we investigate whether participants can refrain from fixating the object that is currently being referred to. If participants can suppress saccades to the referred object, then conclusions that can be drawn from the visual world data would need to be carefully reevaluated.

## EXPERIMENT 1

To test the degree to which conscious eye movement suppression is possible, we manipulated the task in a between-participants visual world experiment: In the free viewing condition, participants were simply instructed to listen to short stories, while in the eye-movement suppression condition, participants were instructed to listen to the stories and avoid looking at the image that the narrator was talking about.

### Methods

#### Participants.

Seventy nine persons took part in the experiment: 40 (aged from 16 to 36 years with the mean of 20; 23 women) received the free viewing task, 39 (age not recorded, but in the similar range; 29 women) received the eye-movement suppression task. All participants were native Russian speakers and took part in the experiment either for course credit or as volunteers. They were tested in the same laboratory setting at the HSE University, Moscow.

The study was carried out in accordance with the ethical principles of psychologists and code of conduct of the American Psychological Association and was approved by the local Institutional Review Board. All participants gave written informed consent in Russian. The study took between 25 and 40 minutes.

#### Materials.

Experimental materials consisted of 64 short stories combined with corresponding visual displays. 32 experimental stories were intermixed with 32 other stories that had similar structure but were not annotated and, subsequently, not analyzed.

**Figure 1.**
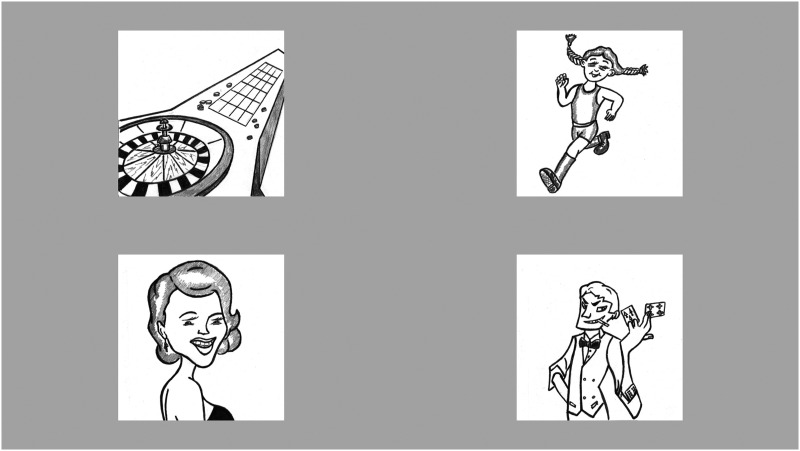
An example of a visual display. All visual displays contained the images of three protagonists (here: actress, athlete, card sharper) and the location (here: casino).

The stories had the same length and described the interactions of three animate protagonists at a certain location (all depicted on the corresponding visual display, see Figure 1), as in Example (1):

(1)  В субботу артистка и спортсменка отчаянно играли в казино на виду у шулера. Наконец артистка обманула спортсменку, чем развеселила шулера. Но он все равно обчистил их обеих в казино. Больше никто не знал, кто спортсменку в субботу обманул в казино.
    On a Saturday night an actress_fem_ and an athlete_fem_ were playing in a casino in front of a card sharper_masc_. At last the actress_fem_ managed to fool the athlete_fem_, which exhilarated the card sharper_masc_. He stripped them both clean in the casino nevertheless. Nobody else knew who fooled the athlete_fem_ in the casino on Saturday night.

The stories and the visual displays remained the same across the two conditions, only the task the participants received varied. No questions were introduced after the stories in order to decrease the pressure to comprehend and create the most favorable circumstances for eye-movement suppression. To preview, we will contrast Experiment 1 with Experiment 2, where comprehension questions were introduced.

Stories that mention multiple protagonists several times showcase a more natural use of language than isolated sentences typically used in the passive listening visual world experiments. Yet, such stimuli are less typical and may raise the question whether any differences between conditions arise due to repeated references to the objects. To evaluate this possibility, we report an additional analysis that includes only eye movements made during the first mention of each object (see Appendix A: Analysis of the First Mention). There was virtually no difference between this subset of data and the whole data set; for this reason, only visual summaries are reported in the Appendix. The statistical analysis of the first mention subset can be found in the online repository. Another important strength of the design is that stories provide a natural context for the use of not only noun-object reference but also pronoun-object reference. In Experiment 2, we will additionally evaluate whether eye-movement suppression depends on the type of referring expression.

The locations of the images on the screen were pseudo-randomized in such a way that protagonists with similar characteristics (agent of the first-mentioned transitive action, etc.) were displayed in different positions across trials. All the black-and-white images were produced by the same illustrator in the same style. The images were presented on a gray background in order to reduce overall screen brightness and make the experiment less taxing for the eyes.

The audio was recorded by a professional female announcer, a native Russian speaker, with an average speed of four syllables per second. For each piece of audio recording, the timing of the start and the end of each noun referring to an image on the screen (see the underlined words in Example 1) was measured in milliseconds based on a spectrogram by a trained linguist, a native speaker of Russian. The time from the onset to the offset of the noun shifted by 200 ms was then used as the analysis time window. The average duration of the nouns comprised 630 (*SD* = 118) ms. Each story had four to ten annotated nouns, with a total of 255 annotated words throughout the experiment. These 255 annotated words consisted of 128 unique nouns, some of which were repeated several times within a story (such as “the athlete” in Example (1)).

#### Procedure.

Images were presented in the four corners of a 24-inch ASUS VG248QE monitor (resolution: 1920 × 1080 px, response time: 1 ms, frame rate: 144 Hz) controlled by a ThinkStation computer. The presentation of the materials and recording of the eye movements were implemented in Experiment Builder (SR Research Ltd.). Participants were tested individually with the Eyelink 1000+ desktop mount eye-tracker using a chin rest. They were seated at a distance of approximately 55 cm from the camera and 90 cm from the monitor. Only the right eye was tracked, at 1000 Hz rate. 9-points calibration was performed before the beginning of the experiment and after a break in the middle of the experiment.

Each trial began with a fixation point in the center of the screen. If the participant fixated it for at least 500 ms, the trial presentation automatically commenced; otherwise, after two seconds, 9-point calibration was repeated. The four images were first presented for one second to allow participants to establish the location of the objects on the screen, and after that, the audio recording of the story was played. Although visual world experiments sometimes employ previews of three seconds and longer (Andersson et al., 2011, Expt. 1; Ferreira et al., 2013, Expts. 1 and 4; Huettig & Guerra, 2019, Expt. 1; Huettig & McQueen, 2007, Expt. 1; Snedeker & Trueswell, 2004; Spivey et al., 2002; Trueswell et al., 1999), previews as short as 300 ms have been shown to provide participants with enough time to extract relevant visual and/or conceptual information associated with the objects on the screen even if the objects were not fixated (Dahan & Tanenhaus, 2005; Dahan et al., 2007; Gardner et al., 2021; Hintz et al., 2017, Expts. 1 and 2; Huettig & Altmann, 2005; Huettig & Guerra, 2019, Expts. 2 and 3; Rommers et al., 2013, Expts. 1 and 3). Even if the present one-second preview is too short, it would make participants look at the objects on the screen more, not less. And if under such circumstances they can still suppress saccades to the referred images in the eye-movement suppression condition, the evidence for the conscious control over eye movements would be even stronger.

In the eye-movement suppression condition, participants received no feedback as to whether they were successful in avoiding looking at the image that was being referred to.

### Analyses

Eye-movement data were split into fixations, saccades, and blinks based on the algorithm from the Data Viewer package (SR Research Ltd). Statistical analysis and data visualization were performed using R (R Core Team, 2016). Data were analyzed using (generalized) linear mixed models [(G)LMMs]. The models were estimated in a Bayesian framework using ‘brms’ package (Bürkner, 2017). The plots were produced with the ‘ggplot2’ and ‘tidybayes’ packages (Kay, 2019; Wickham, 2016). We report effects in terms of 95% credible intervals and the corresponding probability that the estimate is greater than or smaller than zero.

Each model included the fixed effects of the instruction type (eye-movement suppression condition was coded as 1, free viewing as −1), trial number, and the interaction between the trial number and the instruction type (we hypothesized that being further in the experiment may exhaust inhibitory control required to suppress eye movements, but this should happen only in the eye-movement suppression condition). The trial indices were scaled (but not centered) such that predictors could have comparable orders of magnitude. Each model included random intercepts for participants, stories, and individual words, as well as by-story and by-word random slopes for the type of instruction. Including a by-participant random slope for the type of instruction is not possible because instructions varied between participants. Correlations between random slopes were not estimated to facilitate model fitting.

The results are reported in terms of the posterior mean, the 95% percentile intervals (95%-CrI), and the posterior probability of the parameter in question being being greater than zero (*P*(*β* > 0)). Inferences were based on the latter quantity. The cutoff value was specified as 0.975, which corresponds to the critical level of the two-sided *t* test.

The question that the study addresses is whether language-mediated saccades to the referred objects can be suppressed. Since 100% suppression is hardly possible, and any other threshold would be arbitrary, we will not argue for any strict threshold. Instead, we quantify the degree of successful suppression and leave it to the reader to decide whether such degree of suppression constitutes compelling evidence for control of language-mediated eye movements. Recall, for example, that in the study by Salverda and Altmann (2011), participants could refrain from fixating the referred image in 90% of trials. As a reference level, we plot the 5% threshold for the estimated probability of looking at the target image / performing an incoming saccade to the target image.

To quantify whether language-mediated eye movements can be suppressed, we evaluated four dependent measures, which, taken together, should provide a comprehensive picture:

(i)  the probability of the target image (the image depicting the current referent) being fixated during the time window in which the referent is being mentioned, i.e., whether there is at least one fixation on the target image. This measure includes both the cases when the image is already fixated at the start of the time window, and the cases when there is an incoming saccade to the target image. The limitation of this measure is that an image may be fixated at the start of the time window due to both predictive language processing and reasons completely unrelated to language processing;
(ii)  the probability of an incoming saccade to the target image if the image was not fixated at the beginning of the time period. Unlike (i), this measure does not include the cases when the image was fixated throughout the time period. Although both measures (i) and (ii) have their disadvantages, together, they should provide a comprehensive picture of the degree of eye-movement suppression;
(iii)  the time spent fixating the target image in case it was fixated. This measure shows whether participants who could not help fixating the target image would at least try to move their eyes away more quickly. This measure reflects late attempts at conscious eye-movement suppression;
(iv)  individual participants’ estimates of measures (i) and (ii) in order to gauge individual differences in participants’ ability to suppress language-mediated eye movements. The estimates were computed by combining the fixed effects and the individual participants’ random intercepts estimated by the mixed-effects models; these estimates are not empirical means.

Each measure was calculated within the time window corresponding to the sound of each noun (see Procedure) shifted by 200 ms to allow for saccade planning and execution (Matin et al., 1993; Viviani, 1990).

### Results

**Figure 2.**
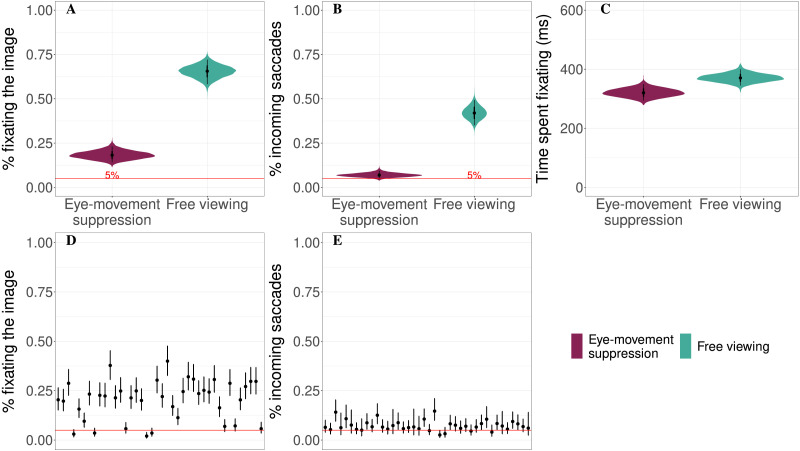
Results of Experiment 1. Panel A: Estimated probability of fixating the target image for each condition. Panel B: Estimated probability of an incoming saccade to the target image when target image was not fixated at the beginning of the time period. Panel C: Time spent fixating the target image if it was fixated. Panels D and E: Individual participants’ probabilities of fixating the target image (D) and making a saccade to the target image (E) estimated for the eye-movement suppression condition. The red line marks the 5% threshold. Violin plots and black lines represent the 95% credible intervals.

In the eye-movement suppression condition, the estimated probability of fixating the target image at least once was lower than in the free viewing condition (18% vs. 66%, the estimated difference between conditions is $\hat{\beta }$ = −47%, 95%-CrI: [−55, −38]%, *P*(*β* < 0) > 0.99; see Figure 2A) and there were fewer incoming saccades (7% vs. 42%, the estimated difference between conditions is $\hat{\beta }$ = −35%, 95%-CrI: [−41, −29]%, *P*(*β* < 0) > 0.99; see Figure 2B). For the subset of data where the target image was fixated, the time spent on the image was shorter in the eye-movement suppression condition (320 vs. 371 ms, the estimated difference between conditions is $\hat{\beta }$ = −50 ms, 95%-CrI: [−88.9, −13] ms, *P*(*β* < 0) > 0.99; see Figure 2C). Individual participants’ estimated probabilities of fixating the target image and making a saccade to it in the eye-movement suppression condition are presented in Figure 2D and Figure 2E. There was no effect of trial or interaction between trial and the instruction type in any of the analyzed measures.

### Discussion

Can language-mediated eye movements be successfully suppressed? Our results suggest that a substantial degree of conscious suppression is possible. A potential objection is that the low proportion of fixations can have two explanations: Either language-mediated eye movements were successfully suppressed or the linguistic input was not processed. We cannot distinguish between these two possibilities because we did not assess comprehension. Having no comprehension questions and, more broadly, no task at all is fairly common in the passive listening studies using visual world paradigm (Altmann & Kamide, 1999, 2007; Huettig & Altmann, 2005; Kamide et al., 2003; Knoeferle & Crocker, 2006; Knoeferle et al., 2005; The ManyBabies Consortium, 2020). It is highly likely that the participants fully processed the linguistic input since at least some components of language processing have been shown to be automatic across many different tasks and paradigms (Humphreys et al., 1982; Pickering & Branigan, 1999; Pickering & Garrod, 2004; Shtyrov & Pulvermüller, 2007; Stroop, 1992; Stupina et al., 2018). However, in order to completely exclude the no-processing explanation for the observed results, the experiment was replicated with added comprehension questions.

## EXPERIMENT 2

Experiment 2 replicated Experiment 1 with the addition of comprehension questions following every story. We describe only the differences between Experiments 1 and 2.

Eighty persons who did not take part in Experiment 1 participated in Experiment 2: 40 participants had the free viewing task, and another 40 participants had the eye-movement suppression task. Unfortunately, demographic information was lost but the sample was very similar to that of Experiment 1. All stories were followed by binary choice comprehension questions. For half of the stories, the correct response was “yes”, and for the other half, “no”. For the example story given in (1), the corresponding question was: “Were the actress and the athlete able to beat the card sharper? Yes / No”. Questions were presented in written form after the end of the trial. To answer the question, participants had to click on the word they chose as the answer. The rest of the experimental materials and procedure remained the same as in Experiment 1.

For Experiment 2, we also report two additional analyses. After determining that a substantial degree of eye-movement suppression is possible, we investigate: i) where participants look when they successfully suppress eye movements to the referred image, and ii) how the degree of eye-movement suppression depends on the referent (one of three animate protagonists or location) and referring expression (noun vs. pronoun). If participants are not consciously aware of pronouns referring to objects in a fashion similar to nouns, they will not try to suppress fixations on the referents of pronouns.

### Results

#### Question Response Accuracy.

The by-participant question response accuracy varied from 65% to 97%, with a mean of 84%. In the eye-movement suppression condition, estimated accuracy was 81%, and in the free viewing condition, 85%. The estimated difference between conditions is $\hat{\beta }$ = −4.4%, 95%-CrI: [−8.1, −0.84]%, *P*(*β* < 0) > 0.99.

We report an additional analysis of eye movements of those participants whose accuracy was above 75% in Appendix B: Analysis of Participants With High Comprehension Question Accuracy. There was little difference between this subset of data and the whole data set; for this reason, only visual summaries are reported in the Appendix. The full analysis can be found in the online repository.

#### Eye Movement Data.

To compare Experiments 1 and 2 directly, the analysis of the pooled data set is presented. The models included the fixed effects of trial, instruction, and comprehension questions (Experiment 2 with comprehension questions was coded as 1, Experiment 1 without questions coded as −1), aswell as the interactions between trial and instruction, and instruction and comprehension questions. The random effects structure included random intercepts for participants, stories, and individual words, as well as by-word and by-story random slopes for the fixed effects of instruction, question, and their interaction. As before, by-participant random slopes could not be included due to the between-participants nature of the design.

**Figure 3.**
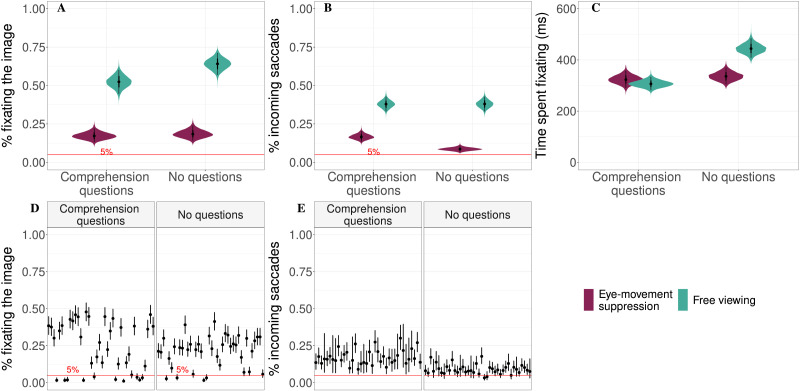
Analysis of the pooled data set from Experiments 1 and 2. Panel A: Estimated probability of fixating the target image for each condition. Panel B: Estimated probability of an incoming saccade to the target image when the target image was not fixated at the beginning of the time period. Panel C: Time spent fixating the target image if it was fixated. Panels D and E: Individual participants’ probabilities of fixating the target image (D) and making a saccade to the target image (E) estimated for the eye-movement suppression condition. The red line marks the 5% threshold. Violin plots and black lines represent the 95% credible intervals.

(i)  **probability of fixating the target image at least once**. In the eye-movement suppression condition, for the pooled data of Experiments 1 and 2, the estimated probability of fixating the target image at least once was lower than in the free viewing condition (18% vs. 58%, the estimated difference between conditions is $\hat{\beta }$ = −41%, 95%-CrI: [−47, −34]%, *P*(*β* < 0) > 0.99; see Figure 3A). Comprehension questions tended to decrease the probability of fixating the target image: 32% vs. 39%, the estimated difference is $\hat{\beta }$ = −6.5%, 95%-CrI: [−13, 0.39]%, *P*(*β* < 0) = 0.97. There was no interaction between the type of instruction and the presence of questions ($\hat{\beta }$ = 4.6%, 95%-CrI: [−2.3, 11]%, *P*(*β* > 0) = 0.91). The probability of fixating the target image decreased over the course of the experiment ($\hat{\beta }$ = −3.6%, 95%-CrI: [−5.9, −1.3]%, *P*(*β* < 0) > 0.99), and this decrease was more pronounced in the eye-movement suppression condition (1% additional decrease, *P*(*β* < 0) = 0.999). This goes against the hypothesized exhausted inhibitory control. Instead, over the course of the experiment participants seem to lose interest in looking at the referred images and thus become better at suppressing language-mediated eye movements. Estimated individual participants’ probabilities of fixating the target image in the eye-movement suppression condition are presented in Figure 3D.
(ii)  **probability of an incoming saccade to the target image**. There were fewer incoming saccades to the target image in the eye-movement suppression condition (12% vs. 38%, the estimated difference is $\hat{\beta }$ = −26%, 95%-CrI: [−30, −22]%, *P*(*β* < 0) > 0.99; see Figure 3B) and in the absence of comprehension questions (19% vs. 26%, the estimated difference is $\hat{\beta }$ = 6.5%, 95%-CrI: [3.3, 9.7]%, *P*(*β* > 0) > 0.99). There was an interaction between the type of instruction and the presence of questions $\hat{\beta }$ = 6.5%, 95%-CrI: [3.4, 9.8]%, *P*(*β* > 0) > 0.99. Within eye-movement suppression conditions, questions increased the probability of an incoming saccade (with questions 17%, without questions 9%, *P*(*β* < 0) = 0.999). Within free-viewing conditions, there was no difference (38% both with and without questions, *P*(*β* < 0) = 0.485). The probability of an incoming saccade decreased over the course of the experiment ($\hat{\beta }$ = −4.1%, 95%-CrI: [−7, −1.3]%, *P*(*β* < 0) > 0.99), and this decrease was more pronounced in the eye-movement suppression condition (1% additional decrease, *P*(*β* < 0) = 0.999). Individual participants’ estimated probabilities of performing an incoming saccade in the eye-movement suppression condition are presented in Figure 3E.
(iii)  **time spent on the target image when the target image was fixated**. Participants fixated the target image for a shorter time in the eye-movement suppression condition (329 ms vs. 369 ms, the estimated difference between conditions is $\hat{\beta }$ = −40 ms, 95%-CrI: [−77, −6.1] ms, *P*(*β* < 0) = 0.99; Figure 3C) and when comprehension questions were asked (315 ms vs. 386 ms, the estimated difference between conditions is $\hat{\beta }$ = −71 ms, 95%-CrI: [−99.9, −45] ms, *P*(*β* < 0) > 0.99). There was an interaction between the type of instruction and comprehension questions ($\hat{\beta }$ = 58 ms, 95%-CrI: [30.1, 85] ms, *P*(*β* > 0) > 0.99): Rather counter-intuitively, within the free viewing condition, comprehension questions decreased the duration of fixating the target image (307 ms vs. 444 ms, *P*(*β* < 0) = 0.999); within the eye-movement suppression condition, there was no difference (336 ms vs. 323 ms, *P*(*β* < 0) = 0.755).

### Discussion

Here, we focus on how comprehension questions affect viewing behavior in the visual world paradigm. Comprehension questions are traditionally viewed as a tool that not only allows the researchers to assess the interpretation of the stimulus, but also directs participants’ attention to the task and ensures deep processing. In the visual world paradigm, one could expect that when comprehension questions promote deep processing, participants would look at the images more and use the images as anchors for interpretation. Counter-intuitively, in the free viewing condition, i.e., in the typical passive listening setting, comprehension questions decreased both the probability of fixating the target image (by 12% with 95%-CrI: [2, 22]%) and the time spent on the target (by 137 ms with 95%-CrI: [99, 179] ms). Possible underlying reasons will be addressed in the General Discussion, but the practical implication is that if researchers are interested in the amount of fixations on the referred images, comprehension questions may reduce the amount of eye movement data available for analysis, which, in turn, reduces statistical power/precision.

### Where Do Participants Look in the Eye-Movement Suppression Condition?

Overall, participants successfully suppressed eye movements to the referred images, at least to some degree. An interesting question here is: Where did they look instead? Two strategies (and any combination thereof) are possible: Participants could either look at some point on the screen other than any image, or engage in active evading, i.e., look at the images, but not at the referred ones. Under the active evading strategy, the time spent fixating each image should be approximately the same across conditions, just distributed differently over time windows. Under the single-point fixation strategy, the time spent fixating the mouse pointer (located in the center of the screen at the beginning of each trial^1^) and the empty space should be greater in the eye-movement suppression condition.

**Figure 4.**
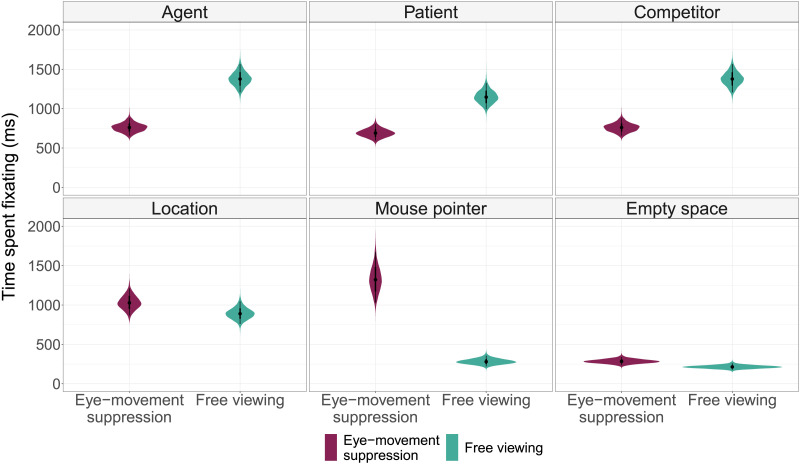
Estimates of total viewing time on various areas of the screen (collapsed across questions/no questions condition). Agent refers to the agent of the first mentioned transitive action (the actress), patient–to the patient of the first-mentioned transitive action (the athlete), and competitor–to the protagonist who was not involved in this action (the card shaper). Violin plots and black lines represent the 95% credible intervals.

We analyzed how long each area on the screen had been fixated during all of the analyzed time windows taken together (here, we do not take into account which image was referred to). Below, we will refer to the story protagonists as agent (of the first-mentioned transitive action, ‘the actress’), patient (of the first-mentioned transitive action, ‘the athlete’), competitor (who was not involved in this action, ‘the card shaper’), and location. A summary of estimated dwell times can be found in Figure 4 (full models can be found in Appendix C: Analysis of the Suppression Strategies). Participants seem to prefer the single-point fixation strategy: In the eye-movement suppression condition, they looked less at the protagonists and more at the empty space and the mouse pointer. Interestingly, there was no difference in the amount of time spent fixating location. Looking at a particular image (mouse pointer) seems to be easier for participants than looking at the empty space on the screen. To further illustrate typical free viewing and single point fixation behaviors, we provide example eye-movement recordings in the online repository (see Data Availability Statement).

This additional analysis also shows that in conditions with comprehension questions, participants not only looked less at the referred image (see Discussion above), they overall looked less at the agent and patient of the first-mentioned action, and at the location, while at the same time looking more at the empty space on the screen instead (see Table C1). In fact, when participants expected to answer comprehension questions, even the average fixation duration and total dwell time on the screen during the whole story were shorter (by 35.4 [10.2, 61.8] ms and 534 [264, 842] ms, respectively).

### Does the Degree of Eye-Movement Suppression Depend on the Referent and Referring Expression?

**Figure 5.**
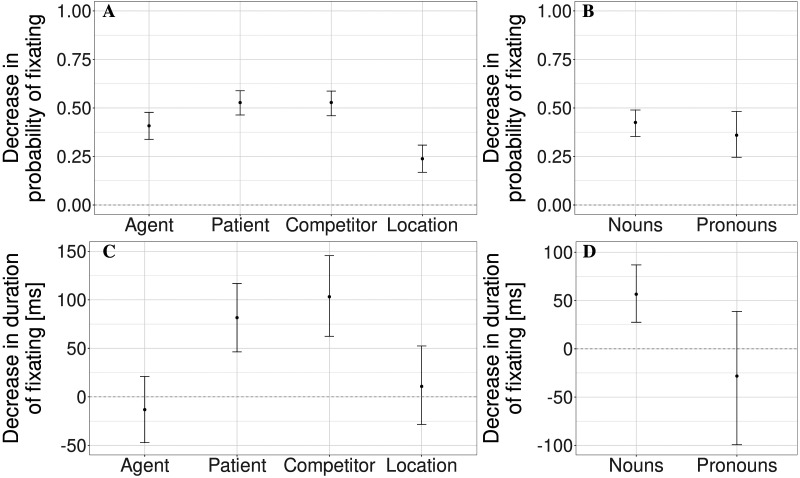
Estimated decrease in probability (panels A, C) or duration (panels B, D) of fixating the referred image depending on the type of referent, referring expression, and experimental condition. Estimates are collapsed across the questions/no questions condition. Positive estimates correspond to decreased probability/time spent fixating the referent. Black lines represent the 95% credible intervals.

The last question we aimed to answer is whether the degree of eye-movement suppression is uniform, or whether fixations on some protagonists were harder to suppress than on others. To this end, we have added the type of referent and all possible interactions with it to the previously fit models estimating probability of fixating the image and the time spent fixating it (measures (i) and (ii)). The resulting estimates collapsed across the question/no question condition are presented in Figures 5A and 5C (for full analysis, see Tables C2 and C3 in Appendix). While probability of fixating each image was lower in the eye-movement suppression condition, the degrees of eye-movement suppression differed. Compared to the agent of the first-mentioned action, the degree of suppression was greater for the patient and the distractor, and lower for the location. Furthermore, when the image was fixated, dwell times did not differ between the suppression and the free-viewing conditions for the agent and the location, but were lower for the patent and the competitor. At present, we can only speculate about the possible reasons for lower eye-movement suppression rates for the agent and location. For location, lower degree of suppression may occur because participants might perceive reference to inanimate objects to be less important than to the animate ones, and therefore, make fewer attempts to suppress their eye movements when location was referred to. Lower recall rates for locative adjuncts (compared to nearly perfect recall of direct objects) reported by Chromý and Vojvodić (2024) support the proposal that participants perceive locations as relatively unimportant. For the agent, the reasons for lower suppression rates are less clear: they may have to do with the given status of the agent, or, on the contrary, with its high level of activation that counteracts suppression strategies.

It is possible that on the whole, noun-object reference was easy for the participants to register and therefore, they could suppress corresponding eye movements successfully. Pronoun-object reference is less straightforward, might be more difficult to register, and may not lead to the same level of conscious eye-movement suppression. To test this, we took advantage of two pronouns present in each story, annotated the time windows corresponding to these pronouns, and the objects these pronouns referred to. The analysis included all three fixed effects (instruction type, presence of questions, and reference type) and all possible interactions between these effects. We found that participants suppressed fixations on the images referred to by nouns and pronouns to a similar degree (see Figure 5B and Table C4). When the image was fixated, dwell times did not differ between the suppression and the free-viewing conditions for the referents of pronouns (see Figure 5D and Table C5). This could be attributed either to shorter duration of an average pronoun or to lower consciousness of fixating the referred image.

## GENERAL DISCUSSION

The goal of the present study was to establish whether, and to what degree, eye movements in the visual world paradigm can be suppressed. The eye-movement suppression task decreased the proportion of fixations on the referred images from 58% (95%-CrI: [53, 64]%) to 18% (95%-CrI: [15, 22]%) across the two experiments, with and without comprehension questions. The probability of making a saccade to the referred image decreased from 38% (95%-CrI: [34, 42]%) to 12% (95%-CrI: [10, 14]%). Comprehension questions did not substantially change the outcomes. Likewise, the degree of suppression was similar for the referents of nouns and pronouns.

To perform the suppression task, on average, participants adopted the single-point fixation strategy: They looked at the mouse pointer in the center of the screen and on the empty space. This mirrors the single-point fixation strategies reported for heritage speakers (Sekerina & Laurinavichyute, 2020; Sekerina & Trueswell, 2011). We will discuss the implications of this viewing behavior for the visual world experiments in the next section.

It is unclear whether the 18% of fixations and 12% of saccades to the target image could be viewed as purely language-mediated eye movements: Even without linguistic input, participants trying to avoid a certain area on the screen would still occasionally fixate it. For example, Salverda and Altmann (2011) report that participants who were instructed to look at the fixation cross in the center of the screen still fixated another image (that was not referred) in 3.9% (Exp. 1) and 6% of trials (Exp. 2). Note that Salverda and Altmann (2011) had only two images on the screen, while this study had four, and the probability of looking at one of these by chance should be even lower. With this in mind, the 18% and 12% probabilities are sufficiently high for us to conclude that most of these eye movements were language-mediated, and participants could not fully suppress language-mediated saccades to the target images. The link between language processing and language-mediated eye movements seems to be difficult to break entirely.

At the same time, our results suggest that eye movements can be decoupled from language processing to a large extent: Even if language processing shifts attention towards the currently referred images, attention can be allocated covertly rather than overtly, as we see here. In particular, language-mediated eye movements can be suppressed to a considerable degree while comprehension is successfully maintained: A major, by 40% (95%-CrI: [34, 47]%), decrease in the probability of fixating the image referred to by either a noun or a pronoun was not accompanied by similarly deteriorated linguistic representations. Although comprehension question accuracy decreased by 4% (95%-CrI: [0.1, 9]%), this decrease is much more moderate compared to the sharp 40% drop.

The present results fit into the goal-oriented accounts of eye movements in the visual world paradigm highlighting the importance of the listener’s goal over looking at the referred objects (Salverda et al., 2011). In what follows, we discuss the potential implications of our findings for some types of visual world designs.

### Potential Implications

The degree of possible decoupling of overt eye movements from language processing may be seen as at odds with one of the main strengths of the visual world paradigm, its perceived ability to show how language processing unfolds in time. Our results suggest that in an extreme case of experimentally prompted eye-movement suppression, the visual world paradigm reflects the ongoing parsing only to a limited extent. This places eye movements closer to a mediated behavioral response, such as grammaticality judgments, than to a more unconscious index of processing, such as ERP or fMRI signals.

What does this mean for the interpretation of the visual world studies? Recall the hypothetical case processing study, in which two groups of participants listen to sentences such as “The bear_NOM_ is watching the rabbit_ACC_” or “The rabbit_ACC_ is watching the bear_NOM_” and look at the corresponding images. If in one group of speakers fixations on the target image occur later and/or do not reach the same maximum frequency as in the other group, can researchers conclude that the underlying processing is slowed down and/or less efficient? On the one hand, since overt eye movements can be to a great degree decoupled from successful language processing, such a conclusion might be unwarranted. After all, participants in the eye-movement suppression condition looked at the images much less than participants in the free-viewing condition, but their language processing was not deficient. On the other hand, there is a fundamental difference between our participants and the group of hypothetically less skilled comprehenders: Participants in the eye-movement suppression condition were actively trying to avoid looking at the target images, while the hypothetically less skilled comprehenders had a traditional free-viewing task.

We suggest that the relative decoupling of eye movements from language processing may matter even for some traditional free-viewing setups due to a combination of two factors. First, most fixations on the target images can be withdrawn: In our study, only about 18% of all fixations made during listening and 12% of saccades are estimated to be unavoidable and automatically language-mediated, the rest could, by assumption, be distributed freely. In other words, participants do not need to look at the referred images a lot in order to maintain successful language processing. They may do so, or they may look elsewhere. Second, and crucially, the option to look elsewhere is likely to be contingent on the experimental design.

The most plausible reasons for not looking at the referred image in the visual world paradigm come down to increased cognitive load. *Gaze aversion* is a long-known response to a cognitively demanding activity: While thinking, people tend to spontaneously look away from the face of an interlocutor or from any potentially distracting stimulus (Doherty-Sneddon et al., 2002; Ehrlichman, 1981; Glenberg et al., 1998). In naturalistic scene viewing, Walter and Bex (2021) report that under increased cognitive load, participants make fewer but longer fixations, and fewer saccades (see also Liu et al., 2022). Our data also supports these observations: In the eye-movement suppression condition, the average fixation duration was 102 [76.9, 125] ms greater, and the overall trial dwell time 424 [125, 716] ms greater than in the free-viewing condition. In the typical visual world studies, high cognitive load could manifest as looking at the single image, the fixation cross, or nowhere in particular in order to spare cognitive resources, much like in our eye-movement suppression condition. Indeed, Sekerina and Trueswell (2011) report that heritage speakers, who experience difficulties with oral language comprehension, systematically look at all the images in a visual world scene much less than native speakers, and keep looking at the fixation cross instead (see also Sekerina & Laurinavichyute, 2020, who report a similarly hesitant viewing pattern in heritage speakers coupled with high accuracy, >90%).

Coming back to our hypothetical example study, it is possible that less skilled comprehenders, who experience greater cognitive load, may look at the fixation cross or the background more, and at the images less even if their language processing is successful in the end. If this is the case, their fixations do not reflect moment-to-moment language processing anymore, which means that nothing can be concluded about language processing speed (for example, speed of lexical access or morphological processing) based on their eye movement data. The same reasoning also holds for within-group comparisons: Participants experiencing higher cognitive load in one experimental condition may make fewer fixations and saccades, or look at the single point on the screen, which would translate to delays in fixating the target image. In fact, Ito et al. (2018) demonstrated that under cognitive load (memorizing a list of five words), the proportion of predictive fixations on the target image decreases by astounding 30%, almost as much as in our eye-movement suppression condition, both in second language learners and native speakers. Importantly, delays in fixating the target image in a setup that creates higher cognitive load do not necessarily reflect delays in language processing.

In the visual world setups where differences in cognitive load between groups or conditions are possible, it might be advisable to test for differences in basic viewing behavior. If there are systematic differences, such as lower number of fixations, longer fixation durations, lower blinking rate, or fewer fixations on the images overall, as expected under increased cognitive load, then researchers may draw conclusions about processing difficulty but not about processing speed. The reasoning behind this check is that if in one condition participants engage in gaze aversion to spare processing resources, then their viewing behavior does not reflect moment-to-moment language comprehension anymore. Conversely, if there are no systematic differences in basic viewing behavior between conditions, researchers can interpret the differences in the timing of fixating the referred image as reflecting the relative speed of processing^2^ but not processing difficulty.

To summarize, we showed that in the visual world setting, eye movements can be suppressed to a large extent under explicit instructions. The relative independence of overt eye movements from language processing potentially limits the inferences that can be drawn from some visual world designs. Decreased or delayed fixations on the referred image can have many causes: While they might result from delayed or deficient language processing, they might also result from increased cognitive load induced by the need to support timely and accurate processing. We tentatively suggest that in those experimental designs where one group of participants may experience greater difficulties, or one condition is harder to process than the other, conclusions might sometimes pertain less to speed and accuracy of language processing, and more to the degree of cognitive load experienced by the participants. This change in interpretation does not undermine the results of previous research: Being able to state that a group of hypothetically less skilled comprehenders, such as second language learners, experiences processing difficulties (instead of delays) is a very informative outcome. In fact, for most research questions, the distinction between processing difficulties and delays would not matter in practice. A situation where it might be of importance is, for example, an empirical test of a computational cognitive model that predicts a particular processing slowdown.

## CONCLUSION

This paper aimed to quantify how much eye movements depend on task in the visual world paradigm in the extreme case of eye movement suppression. We show that it is possible to consciously suppress saccades to the images referred to by nouns and pronouns to a great degree: In particular, it is possible to fixate the referred images in 18% of cases on average, and for some individuals, in less than 5% of cases, while still maintaining accurate comprehension (as measured by simple yes/no questions). Participants approached the task strategically: instead of the images, in the eye-movement suppression condition, they looked more at the mouse cursor and empty areas on the screen.

If successful comprehension is not necessarily accompanied by concurrent eye movements, then delayed fixations and/or lower prevalence of fixations on the referred image might not necessarily reflect speed or accuracy of language processing. For the visual world designs where one condition is noticeably harder to process, or one group of participants is expected to have processing difficulties, we tentatively propose that eye movement data may at least sometimes reflect rather the degree of cognitive load experienced by participants than processing speed and/or accuracy. Overall, the present study highlights the need to develop more nuanced accounts of the mechanism that guides eye movements in response to linguistic input, taking into account the listener’s goal and degree of cognitive load.

## ACKNOWLEDGMENTS

The authors thank Tatiana Bolgina for asking the question that started it all, Olga Dragoy for pointing out the possible strategic nature of eye movements, as well as Irina Sekerina, Maximilian Rabe, Dorothea Pregla, Jessica Brown, Dario Paape, Sol Lago, and Camilo Rodriguez Ronderos for their helpful feedback on the previous versions of this manuscript.

## FUNDING INFORMATION

ALau was funded by the Deutsche Forschungsgemeinschaft (DFG, German Research Foundation) – Project-ID 317633480 – SFB 1287.

## AUTHOR CONTRIBUTIONS

Project was formulated by ALau. AZ collected the experimental data. ALop and AZ prepared data for analysis. ALau analyzed the data and wrote the manuscript, ALop provided feedback on the drafts.

## DATA AVAILABILITY STATEMENT

The data and the code for all the reported analyses are available from the project page at the Open Science Framework: https://osf.io/9qzns/.

## Notes

^1^ Participants were free to move the mouse pointer but never did so because during the story, clicking on any object on the screen was not required. In Experiment 2, participants had to click on the correct response to the written questions, but before they saw the question, moving the mouse pointer made no practical sense. Thus, the mouse pointer stayed in the center of the screen throughout the experiment.^2^ But not necessarily the absolute speed, see McMurray (2023).

## Figures and Tables

**Figure A1.**
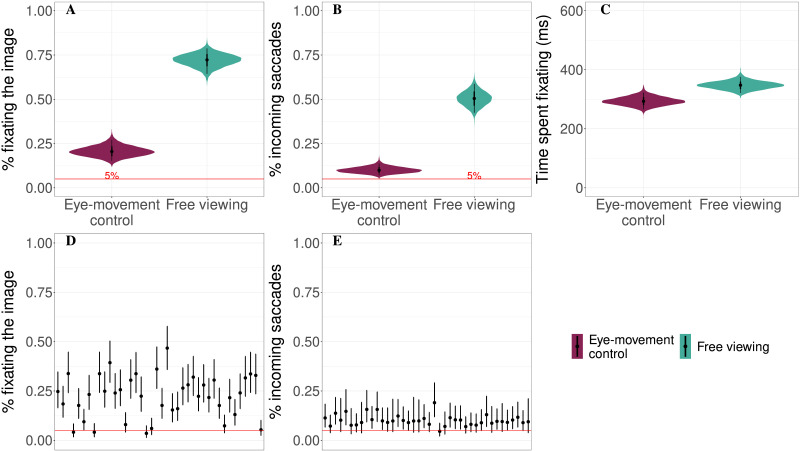
Results of Experiment 1 on the subset of data that includes only the first mention of each object. Panel A: Estimated probability of fixating the target image for each condition. Panel B: Estimated probability of an incoming saccade to the target image when target image was not fixated at the beginning of the time period. Panel C: Time spent fixating the target image if it was fixated. Panels D and E: Individual participants’ probabilities of fixating the target image (D) and making a saccade to the target image (E) estimated for the eye-movement suppression condition. The red line marks the 5% threshold. Violin plots and black lines represent the 95% credible intervals.

**Figure A2.**
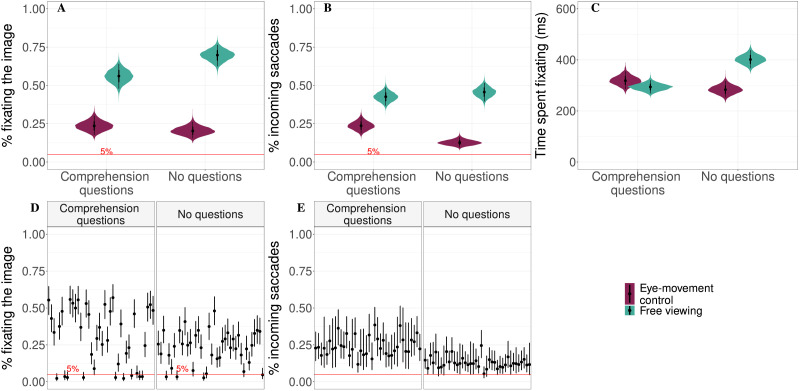
Analysis of the pooled data set from Experiments 1 and 2 that includes only the first mention of each object. Panel A: Estimated probability of fixating the target image for each condition. Panel B: Estimated probability of an incoming saccade to the target image when the target image was not fixated at the beginning of the time period. Panel C: Time spent fixating the target image if it was fixated. Panels D and E: Individual participants’ probabilities of fixating the target image (D) and making a saccade to the target image (E) estimated for the eye-movement suppression condition. The red line marks the 5% threshold. Violin plots and black lines represent the 95% credible intervals.

**Figure B1.**
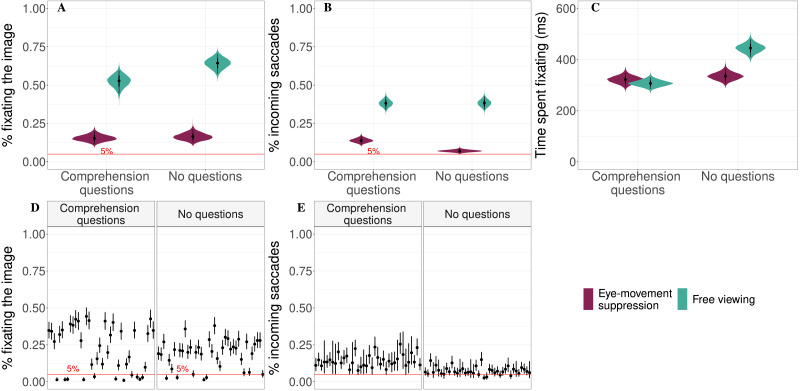
Analysis of the pooled data set from Experiments 1 and 2 that includes only participants with the average accuracy above 75%. Panel A: Estimated probability of fixating the target image for each condition. Panel B: Estimated probability of an incoming saccade to the target image when the target image was not fixated at the beginning of the time period. Panel C: Time spent fixating the target image if it was fixated. Panels D and E: Individual participants’ probabilities of fixating the target image (D) and making a saccade to the target image (E) estimated for the eye-movement suppression condition. The red line marks the 5% threshold. Violin plots and black lines represent the 95% credible intervals.

## APPENDIX A: ANALYSIS OF THE FIRST MENTION

Here, only the subset of data with the first mention of each object in each story is analyzed: Figure A1 shows the results of Experiment 1, and Figure A2–the results of joint data set from Experiments 1 and 2.

## APPENDIX B: PARTICIPANTS WITH HIGH COMPREHENSION QUESTION ACCURACY

Figure B1 presents a visual summary of the analysis of the joint data set that includes participants with the average accuracy above 75%. The details of the statistical analysis can be found in the online repository associated with the paper (see Data Availability Statement).

## APPENDIX C: ANALYSIS OF THE SUPPRESSION STRATEGIES

Table C1 reports where on the screen and for how long participants looked depending on experimental condition. For this analysis, fixations on the same area made during all the analyzed time windows, including the time windows during which this image was not referred to, were added together.

**Table C1.** Sum of fixations on varios areas of the screen during all time windows.

**Agent**	Estimate (log-ms)	95%-CrI
Intercept	6.77	[6.65; 6.89]
Suppression	−0.25	[−0.32; −0.18]
Questions	−0.10	[−0.17; −0.03]
Suppression × Questions	−0.08	[−0.15; −0.02]
**Patient**		
Intercept	6.79	[6.66, 6.92]
Suppression	−0.25	[−0.33; −0.18]
Questions	−0.11	[−0.18; −0.04]
Suppression × Questions	−0.12	[−0.19; −0.04]
**Competitor**		
Intercept	6.93	[6.81, 7.04]
Suppression	−0.30	[−0.37; −0.22]
Questions	−0.02	[−0.10; 0.05]
Suppression × Questions	0.01	[−0.07; 0.08]
**Location**		
Intercept	6.86	[6.73; 6.99]
Suppression	0.07	[−0.03; 0.17]
Questions	−0.14	[−0.24; −0.04]
Suppression × Questions	−0.07	[−0.17; 0.04]
**Mouse pointer**		
Intercept	6.41	[6.23; 6.58]
Suppression	0.77	[0.61; 0.93]
Questions	−0.03	[−0.19; 0.13]
Suppression × Questions	0.10	[−0.06; 0.27]
**The rest of the screen**		
Intercept	5.51	[5.37; 5.64]
Suppression	0.14	[0.01; 0.27]
Questions	0.21	[0.09; 0.34]
Suppression × Questions	0.01	[−0.12; 0.13]

Table C2 presents the analysis of the probability of fixating the referred image depending on the experimental condition and the type of referent; Table C3 reports for how long the image was fixated, if it was fixated.

**Table C2.** Probability of fixating the referred image depending on the experimental conditions and the image referent.

Predictor	Estimate (log-odds)	95%-CrI
Intercept	−0.66	[−0.81; −0.50]
Suppression	−0.96	[−1.13; −0.78]
Questions	−0.18	[−0.33; −0.03]
Patient	0.17	[0.06; 0.28]
Competitor	0.21	[0.09; 0.32]
Location	−0.36	[−0.46; −0.25]
Suppression × Questions	0.07	[−0.09; 0.23]
Suppression × Patient	−0.28	[−0.41; −0.15]
Suppression × Competitor	−0.27	[−0.40; −0.13]
Suppression × Location	0.34	[0.21; 0.48]
Questions × Patient	0.10	[0.04; 0.16]
Questions × Competitor	0.15	[0.09; 0.22]
Questions × Location	−0.04	[−0.10; 0.01]
Suppression × Question × Patient	0.05	[−0.01; 0.11]
Suppression × Question × Competitor	0.11	[0.05; 0.17]
Suppression × Question × Location	−0.02	[−0.08; 0.03]

**Table C3.** Duration of fixating the referred image depending on the experimental conditions and the image referent.

Predictor	Estimate (log-ms)	95%-CrI
Intercept	5.78	[5.72; 5.84]
Suppression	0.02	[−0.03; 0.07]
Questions	−0.11	[−0.15; −0.06]
Patient	−0.00	[−0.07; 0.06]
Competitor	0.14	[0.07; 0.21]
Location	0.15	[0.09; 0.22]
Suppression × Questions	0.08	[0.04; 0.13]
Suppression × Patient	−0.15	[−0.21; −0.09]
Suppression × Competitor	−0.16	[−0.22; −0.10]
Suppression × Location	−0.04	[−0.09; 0.02]
Questions × Patient	−0.01	[−0.06; 0.03]
Questions × Competitor	0.06	[0.02; 0.11]
Questions × Location	−0.03	[−0.07; 0.02]
Suppression × Question × Patient	−0.01	[−0.06; 0.04]
Suppression × Question × Competitor	−0.01	[−0.05; 0.04]
Suppression × Question × Location	0.03	[−0.01; 0.08]

Table C4 presents the analysis of the probability of fixating the referred image depending on the experimental condition and the referring expression; Table C5 reports for how long the image was fixated, if it was fixated.

**Table C4.** Estimated probability of fixating a region depending on the experimental conditions and the type of referring expression.

Predictor	Estimate (log-odds)	95%-CrI
Intercept	−0.95	[−1.14; −0.76]
Suppression	−1.03	[−1.26; −0.82]
Questions	−0.18	[−0.34; −0.02]
Pronouns	0.03	[−0.14; 0.19]
Suppression × Pronouns	−0.03	[−0.19; 0.13]
Questions × Pronouns	−0.05	[−0.10; −0.00]
Suppression × Questions × Pronouns	−0.08	[−0.14; −0.02]

**Table C5.** Estimated duration of fixating a region depending on the experimental conditions and the type of referring expression.

Predictor	Estimate (log-ms)	95%-CrI
Intercept	5.82	[5.76; 5.89]
Suppression	−0.02	[−0.08; 0.05]
Questions	−0.12	[−0.16; −0.07]
Pronouns	0.04	[−0.01; 0.08]
Suppression × Pronouns	0.06	[0.01; 0.11]
Questions × Pronouns	−0.00	[−0.04; 0.03]
Suppression × Questions × Pronouns	−0.04	[−0.06; −0.01]

## References

[bib1] Altmann, G. T. M., & Kamide, Y. (1999). Incremental interpretation at verbs: Restricting the domain of subsequent reference. Cognition, 73(3), 247–264. 10.1016/S0010-0277(99)00059-1, 10585516

[bib2] Altmann, G. T. M., & Kamide, Y. (2007). The real-time mediation of visual attention by language and world knowledge: Linking anticipatory (and other) eye movements to linguistic processing. Journal of Memory and Language, 57(4), 502–518. 10.1016/j.jml.2006.12.004

[bib3] Andersson, R., Ferreira, F., & Henderson, J. M. (2011). I see what you’re saying: The integration of complex speech and scenes during language comprehension. Acta Psychologica, 137(2), 208–216. 10.1016/j.actpsy.2011.01.007, 21303711

[bib4] Batterink, L., & Neville, H. J. (2013). The human brain processes syntax in the absence of conscious awareness. Journal of Neuroscience, 33(19), 8528–8533. 10.1523/JNEUROSCI.0618-13.2013, 23658189 PMC3720232

[bib5] Bürkner, P.-C. (2017). brms: An R package for Bayesian multilevel models using Stan. Journal of Statistical Software, 80(1), 1–28. 10.18637/jss.v080.i01

[bib6] Carrasco, M. (2011). Visual attention: The past 25 years. Vision Research, 51(13), 1484–1525. 10.1016/j.visres.2011.04.012, 21549742 PMC3390154

[bib7] Chromý, J., & Vojvodić, S. (2024). When and where did it happen? Systematic differences in recall of core and optional sentence information. Quarterly Journal of Experimental Psychology, 77(1), 111–132. 10.1177/17470218231159190, 36786323 PMC10712210

[bib8] Cooper, R. M. (1974). The control of eye fixation by the meaning of spoken language: A new methodology for the real-time investigation of speech perception, memory, and language processing. Cognitive Psychology, 6(1), 84–107. 10.1016/0010-0285(74)90005-X

[bib9] Dahan, D., & Tanenhaus, M. K. (2005). Looking at the rope when looking for the snake: Conceptually mediated eye movements during spoken-word recognition. Psychonomic Bulletin & Review, 12(3), 453–459. 10.3758/BF03193787, 16235628

[bib10] Dahan, D., Tanenhaus, M. K., & Salverda, A. P. (2007). The influence of visual processing on phonetically driven saccades in the “visual world” paradigm. In R. P. G. Van Gompel, M. H. Fischer, W. S. Murray, & R. L. Hill (Eds.), Eye movements: A window on mind and brain (pp. 471–486). Elsevier. 10.1016/B978-008044980-7/50023-9

[bib11] DeAngelus, M., & Pelz, J. B. (2009). Top-down control of eye movements: Yarbus revisited. Visual Cognition, 17(6–7), 790–811. 10.1080/13506280902793843

[bib12] Degen, J., Kursat, L., & Leigh, D. D. (2021). Seeing is believing: Testing an explicit linking assumption for visual world eye-tracking in psycholinguistics. In Proceedings of the Annual Meeting of the Cognitive Science Society (Vol. 43, pp. 1500–1506). Cognitive Science Society.

[bib13] Doherty-Sneddon, G., Bruce, V., Bonner, L., Longbotham, S., & Doyle, C. (2002). Development of gaze aversion as disengagement from visual information. Developmental Psychology, 38(3), 438–445. 10.1037/0012-1649.38.3.438, 12005386

[bib14] Eberhard, K. M., Spivey-Knowlton, M. J., Sedivy, J. C., & Tanenhaus, M. K. (1995). Eye movements as a window into real-time spoken language comprehension in natural contexts. Journal of Psycholinguistic Research, 24(6), 409–436. 10.1007/BF02143160, 8531168

[bib15] Ehrlichman, H. (1981). From gaze aversion to eye-movement suppression: An investigation of the cognitive interference explanation of gaze patterns during conversation. British Journal of Social Psychology, 20(4), 233–241. 10.1111/j.2044-8309.1981.tb00492.x

[bib16] Engbert, R., Longtin, A., & Kliegl, R. (2002). A dynamical model of saccade generation in reading based on spatially distributed lexical processing. Vision Research, 42(5), 621–636. 10.1016/S0042-6989(01)00301-7, 11853779

[bib17] Engbert, R., Nuthmann, A., Richter, E. M., & Kliegl, R. (2005). SWIFT: A dynamical model of saccade generation during reading. Psychological Review, 112(4), 777–813. 10.1037/0033-295X.112.4.777, 16262468

[bib18] Falandays, J. B., Brown-Schmidt, S., & Toscano, J. C. (2020). Long-lasting gradient activation of referents during spoken language processing. Journal of Memory and Language, 112. 104088. 10.1016/j.jml.2020.104088

[bib19] Ferreira, F., Foucart, A., & Engelhardt, P. E. (2013). Language processing in the visual world: Effects of preview, visual complexity, and prediction. Journal of Memory and Language, 69(3), 165–182. 10.1016/j.jml.2013.06.001

[bib20] Gardner, B., Dix, S., Lawrence, R., Morgan, C., Sullivan, A., & Kurumada, C. (2021). Online pragmatic interpretations of scalar adjectives are affected by perceived speaker reliability. PLoS One, 16(2), e0245130. 10.1371/journal.pone.0245130, 33606683 PMC7895354

[bib21] Glenberg, A. M., Schroeder, J. L., & Robertson, D. A. (1998). Averting the gaze disengages the environment and facilitates remembering. Memory & Cognition, 26(4), 651–658. 10.3758/BF03211385, 9701957

[bib22] Hayhoe, M., & Ballard, D. (2005). Eye movements in natural behavior. Trends in Cognitive Sciences, 9(4), 188–194. 10.1016/j.tics.2005.02.009, 15808501

[bib23] Hintz, F., Meyer, A. S., & Huettig, F. (2017). Predictors of verb-mediated anticipatory eye movements in the visual world. Journal of Experimental Psychology: Learning, Memory, and Cognition, 43(9), 1352–1374. 10.1037/xlm0000388, 28287762

[bib24] Huettig, F., & Altmann, G. T. M. (2005). Word meaning and the control of eye fixation: Semantic competitor effects and the visual world paradigm. Cognition, 96(1), B23–B32. 10.1016/j.cognition.2004.10.003, 15833303

[bib25] Huettig, F., & Guerra, E. (2019). Effects of speech rate, preview time of visual context, and participant instructions reveal strong limits on prediction in language processing. Brain Research, 1706, 196–208. 10.1016/j.brainres.2018.11.013, 30439351

[bib26] Huettig, F., & McQueen, J. M. (2007). The tug of war between phonological, semantic and shape information in language-mediated visual search. Journal of Memory and Language, 57(4), 460–482. 10.1016/j.jml.2007.02.001

[bib27] Humphreys, G. W., Evett, L. J., & Taylor, D. E. (1982). Automatic phonological priming in visual word recognition. Memory & Cognition, 10(6), 576–590. 10.3758/BF03202440, 7162419

[bib28] Ito, A., Corley, M., & Pickering, M. J. (2018). A cognitive load delays predictive eye movements similarly during L1 and L2 comprehension. Bilingualism: Language and Cognition, 21(2), 251–264. 10.1017/S1366728917000050

[bib29] Kamide, Y., Altmann, G. T. M., & Haywood, S. L. (2003). The time-course of prediction in incremental sentence processing: Evidence from anticipatory eye movements. Journal of Memory and Language, 49(1), 133–156. 10.1016/S0749-596X(03)00023-8

[bib30] Kay, M. (2019). tidybayes: Tidy data and geoms for Bayesian models. Retrieved from https://mjskay.github.io/tidybayes/ (R package version 1.1.0). 10.5281/zenodo.1308151

[bib31] Knoeferle, P., & Crocker, M. W. (2006). The coordinated interplay of scene, utterance, and world knowledge: Evidence from eye tracking. Cognitive Science, 30(3), 481–529. 10.1207/s15516709cog0000_65, 21702823

[bib32] Knoeferle, P., Crocker, M. W., Scheepers, C., & Pickering, M. J. (2005). The influence of the immediate visual context on incremental thematic role-assignment: Evidence from eye-movements in depicted events. Cognition, 95(1), 95–127. 10.1016/j.cognition.2004.03.002, 15629475

[bib33] Knoeferle, P., & Guerra, E. (2016). Visually situated language comprehension. Language and Linguistics Compass, 10(2), 66–82. 10.1111/lnc3.12177

[bib34] Liu, J.-C., Li, K.-A., Yeh, S.-L., & Chien, S.-Y. (2022). Assessing perceptual load and cognitive load by fixation-related information of eye movements. Sensors, 22(3), 1187. 10.3390/s22031187, 35161930 PMC8839381

[bib35] Matin, E., Shao, K. C., & Boff, K. R. (1993). Saccadic overhead: Information-processing time with and without saccades. Perception & Psychophysics, 53(4), 372–380. 10.3758/BF03206780, 8483701

[bib36] McLaughlin, J., Osterhout, L., & Kim, A. (2004). Neural correlates of second-language word learning: Minimal instruction produces rapid change. Nature Neuroscience, 7(7), 703–704. 10.1038/nn1264, 15195094

[bib37] McMurray, B. (2023). I’m not sure that curve means what you think it means: Toward a [more] realistic understanding of the role of eye-movement generation in the Visual World Paradigm. Psychonomic Bulletin & Review, 30(1), 102–146. 10.3758/s13423-022-02143-8, 35962241 PMC10964151

[bib38] Mei, N., Santana, R., & Soto, D. (2022). Informative neural representations of unseen contents during higher-order processing in human brains and deep artificial networks. Nature Human Behaviour, 6(5), 720–731. 10.1038/s41562-021-01274-7, 35115676

[bib39] Pickering, M. J., & Branigan, H. P. (1999). Syntactic priming in language production. Trends in Cognitive Sciences, 3(4), 136–141. 10.1016/S1364-6613(99)01293-0, 10322467

[bib40] Pickering, M. J., & Garrod, S. (2004). Toward a mechanistic psychology of dialogue. Behavioral and Brain Sciences, 27(2), 169–190. 10.1017/S0140525X04000056, 15595235

[bib41] R Core Team. (2016). R: A language and environment for statistical computing. R Foundation for Statistical Computing. https://www.R-project.org/

[bib42] Reichle, E. D., Warren, T., & McConnell, K. (2009). Using E-Z reader to model the effects of higher level language processing on eye movements during reading. Psychonomic Bulletin & Review, 16(1), 1–21. 10.3758/PBR.16.1.1, 19145006 PMC2629133

[bib43] Reilly, R. G., & Radach, R. (2003). Foundations of an interactive activation model of eye movement control in reading. In J. Hyönä, R. Radach, & H. Deubel (Eds.), The mind’s eye: Cognitive and applied aspects of eye movement research (pp. 429–455). Elsevier. 10.1016/B978-044451020-4/50024-4

[bib44] Rohaut, B., & Naccache, L. (2017). Disentangling conscious from unconscious cognitive processing with event-related EEG potentials. Revue Neurologique, 173(7–8), 521–528. 10.1016/j.neurol.2017.08.001, 28843414

[bib45] Rommers, J., Meyer, A. S., Praamstra, P., & Huettig, F. (2013). The contents of predictions in sentence comprehension: Activation of the shape of objects before they are referred to. Neuropsychologia, 51(3), 437–447. 10.1016/j.neuropsychologia.2012.12.002, 23238371

[bib46] Salverda, A. P., & Altmann, G. T. M. (2011). Attentional capture of objects referred to by spoken language. Journal of Experimental Psychology: Human Perception and Performance, 37(4), 1122–1133. 10.1037/a0023101, 21517215 PMC3145002

[bib47] Salverda, A. P., Brown, M., & Tanenhaus, M. K. (2011). A goal-based perspective on eye movements in visual world studies. Acta Psychologica, 137(2), 172–180. 10.1016/j.actpsy.2010.09.010, 21067708 PMC3109199

[bib48] Sekerina, I. A., & Laurinavichyute, A. K. (2020). Heritage speakers can actively shape not only their grammar but also their processing. Bilingualism: Language and Cognition, 23(1), 43–45. 10.1017/S1366728919000440

[bib49] Sekerina, I. A., Laurinavichyute, A. K., & Dragoy, O. (2019). What eye movements can and cannot tell us about *wh*-movement and scrambling. In K. Carlson, C. Clifton, Jr., & J. D. Fodor (Eds.), Grammatical approaches to language processing: Essays in honor of Lyn Frazier (pp. 147–165). Springer. 10.1007/978-3-030-01563-3_8

[bib50] Sekerina, I. A., & Trueswell, J. C. (2011). Processing of contrastiveness by heritage Russian bilinguals. Bilingualism: Language and Cognition, 14(3), 280–300. 10.1017/S1366728910000337

[bib51] Shtyrov, Y., & Pulvermüller, F. (2007). Early MEG activation dynamics in the left temporal and inferior frontal cortex reflect semantic context integration. Journal of Cognitive Neuroscience, 19(10), 1633–1642. 10.1162/jocn.2007.19.10.1633, 17854281

[bib52] Snedeker, J., & Trueswell, J. C. (2004). The developing constraints on parsing decisions: The role of lexical-biases and referential scenes in child and adult sentence processing. Cognitive Psychology, 49(3), 238–299. 10.1016/j.cogpsych.2004.03.001, 15342261

[bib53] Snell, J., van Leipsig, S., Grainger, J., & Meeter, M. (2018). OB1-reader: A model of word recognition and eye movements in text reading. Psychological Review, 125(6), 969–984. 10.1037/rev0000119, 30080066

[bib54] Soto, D., & Humphreys, G. W. (2007). Automatic guidance of visual attention from verbal working memory. Journal of Experimental Psychology: Human Perception and Performance, 33(3), 730–737. 10.1037/0096-1523.33.3.730, 17563233

[bib55] Spivey, M. J., Tanenhaus, M. K., Eberhard, K. M., & Sedivy, J. C. (2002). Eye movements and spoken language comprehension: Effects of visual context on syntactic ambiguity resolution. Cognitive Psychology, 45(4), 447–481. 10.1016/S0010-0285(02)00503-0, 12480476

[bib56] Stroop, J. R. (1992). Studies of interference in serial verbal reactions. Journal of Experimental Psychology: General, 121(1), 15–23. 10.1037/0096-3445.121.1.15

[bib57] Stupina, E., Myachykov, A., & Shtyrov, Y. (2018). Automatic lexical access in visual modality: Eye-tracking evidence. Frontiers in Psychology, 9, 1847. 10.3389/fpsyg.2018.01847, 30333775 PMC6176043

[bib58] The ManyBabies Consortium. (2020). Quantifying sources of variability in infancy research using the infant-directed-speech preference. Advances in Methods and Practices in Psychological Science, 3(1), 24–52. 10.1177/2515245919900809

[bib59] Tokowicz, N., & MacWhinney, B. (2005). Implicit and explicit measures of sensitivity to violations in second language grammar: An event-related potential investigation. Studies in Second Language Acquisition, 27(2), 173–204. 10.1017/S0272263105050102

[bib60] Trueswell, J. C., Sekerina, I., Hill, N. M., & Logrip, M. L. (1999). The kindergarten-path effect: Studying on-line sentence processing in young children. Cognition, 73(2), 89–134. 10.1016/S0010-0277(99)00032-3, 10580160

[bib61] van Gaal, S., Naccache, L., Meuwese, J. D. I., van Loon, A. M., Leighton, A. H., Cohen, L., & Dehaene, S. (2014). Can the meaning of multiple words be integrated unconsciously? Philosophical Transactions of the Royal Society B: Biological Sciences, 369(1641), 20130212. 10.1098/rstb.2013.0212, 24639583 PMC3965166

[bib62] Viviani, P. (1990). Eye movements in visual search: Cognitive, perceptual, and motor control aspects. In E. Kowler (Ed.), Eye movements and their role in visual and cognitive processes (pp. 353–393). Elsevier. 7492533

[bib63] Walter, K., & Bex, P. (2021). Cognitive load influences oculomotor behavior in natural scenes. Scientific Reports, 11(1), 12405. 10.1038/s41598-021-91845-5, 34117336 PMC8196072

[bib64] Wickham, H. (2016). ggplot2: Elegant graphics for data analysis. Springer. 10.1007/978-3-319-24277-4

[bib65] Yarbus, A. L. (1967). Eye movements during perception of complex objects. In Eye movements and vision (pp. 171–211). Springer. 10.1007/978-1-4899-5379-7_8

[bib66] Yoon, S. O., & Brown-Schmidt, S. (2018). Influence of the historical discourse record on language processing in dialogue. Discourse Processes, 55(1), 31–46. 10.1080/0163853X.2016.1193429

[bib67] Zhao, M., Gersch, T. M., Schnitzer, B. S., Dosher, B. A., & Kowler, E. (2012). Eye movements and attention: The role of pre-saccadic shifts of attention in perception, memory and the control of saccades. Vision Research, 74, 40–60. 10.1016/j.visres.2012.06.017, 22809798 PMC3623695

